# Anti-glomerular basement membrane disease mediated by IgG and IgA: a case report

**DOI:** 10.1080/0886022X.2021.1914658

**Published:** 2021-04-29

**Authors:** Guming Zou, Haitao Lu, Li Zhuo, Wanzhong Zou, Wenge Li

**Affiliations:** a Department of Nephrology, China-Japan Friendship Hospital, Beijing, People’s Republic of China; b Institute of Nephrology, Peking University, Beijing, People’s Republic of China

**Keywords:** Anti-glomerular basement membrane (anti-GBM) disease, immunoglobulin G (IgG), immunoglobulin A (IgA), α3(IV) collagen chain

## Abstract

**Background:**

Anti-glomerular basement membrane (anti-GBM) disease is a rare autoimmune condition responsible for rapidly progressive glomerulonephritis. This disease is usually mediated by IgG autoantibodies against the noncollagenous domain of the α3(IV) collagen chain. In rare cases, IgA or IgM anti-GBM antibodies are involved. This raises the question of whether there are different types of antibody-mediated anti-GBM disease at the same time.

**Case report:**

A 37-year-old woman with anti-GBM disease mediated by IgG and IgA. The patient developed rapidly progressive glomerulonephritis with nephrotic syndrome. Indirect immunofluorescence analysis indicated the presence of IgG and IgA antibodies reactive with a basement membrane component, identified by enzyme-linked immunoadsorbent assay and Western blotting as the α3(IV) collagen chain. After plasmapheresis and immunotherapy (steroids and cyclophosphamide), much improved the massive proteinuria and renal function. Follow up to date, she had normal renal function without proteinuria.

**Conclusions:**

This is the first case report of anti-GBM disease mediated by IgG and IgA. If the clinical presentation and histopathological findings are suggestive of atypical anti-GBM disease, alternative laboratory tests such as Western blotting analysis can be used to confirm the diagnosis.

## Introduction

Anti-glomerular basement membrane (anti-GBM) disease is a rare autoimmune condition responsible for rapidly progressive glomerulonephritis, which is mediated by circulating autoantibodies. The principal autoantigen is the α345 network of collagen IV, expression of which is restricted to target tissues [1]. Typically, linear deposition of immunoglobulin along the glomerular basement membrane (GBM) is seen. In most cases, the causative antibody is IgG [2,3]. Rarely, however, anti-GBM disease is mediated by immunoglobulin A (IgA) or immunolgobulin M (IgM) antibodies [4–7]. Here, we describe a new case of anti-GBM disease mediated by immunolgobulin G (IgG) and IgA.

## Case report

A 37-year-old woman was admitted to the renal unit for rapidly progressive glomerulonephritis. Medical history included fracture of the left clavicle 10 years ago. She has no smoking or alcohol, no history of hydrocarbon exposure, chemicals or heavy metal exposure, etc.

The results of physical examination on admission, including blood pressure (110/65 mmHg), were normal. Serum creatinine had risen from 72 μmol/L to 310 μmol/L over the past 2 weeks. Urinary examination disclosed microscopic hematuria and proteinuria within the nephrotic range (8.11 g/day). She had hypoproteinemia (albumin 24 g/L) with no anemia (hemoglobin 124 g/L). Routine ELISAs were positive for anti-GBM (104 RU/mL, normal range <20 RU/mL) (EA 1251-9601 G; Euroimmun Medizinische Labordiagnostika (China), Beijing, China). Tests for IgA anti-GBM were not available on admission. Other laboratory investigations showed normal C3 and C4 levels and negative anti-nuclear antibody, anti-phospholipid antibody, and anti-neutrophil cytoplasmic antibody (ANCA). Immunoglobulins G, M, and A were normal and no paraprotein was found in serum or urine. Ultrasound showed normal kidneys. Computed tomography (CT) excluded alveolar hemorrhage.

**Figure 1.**
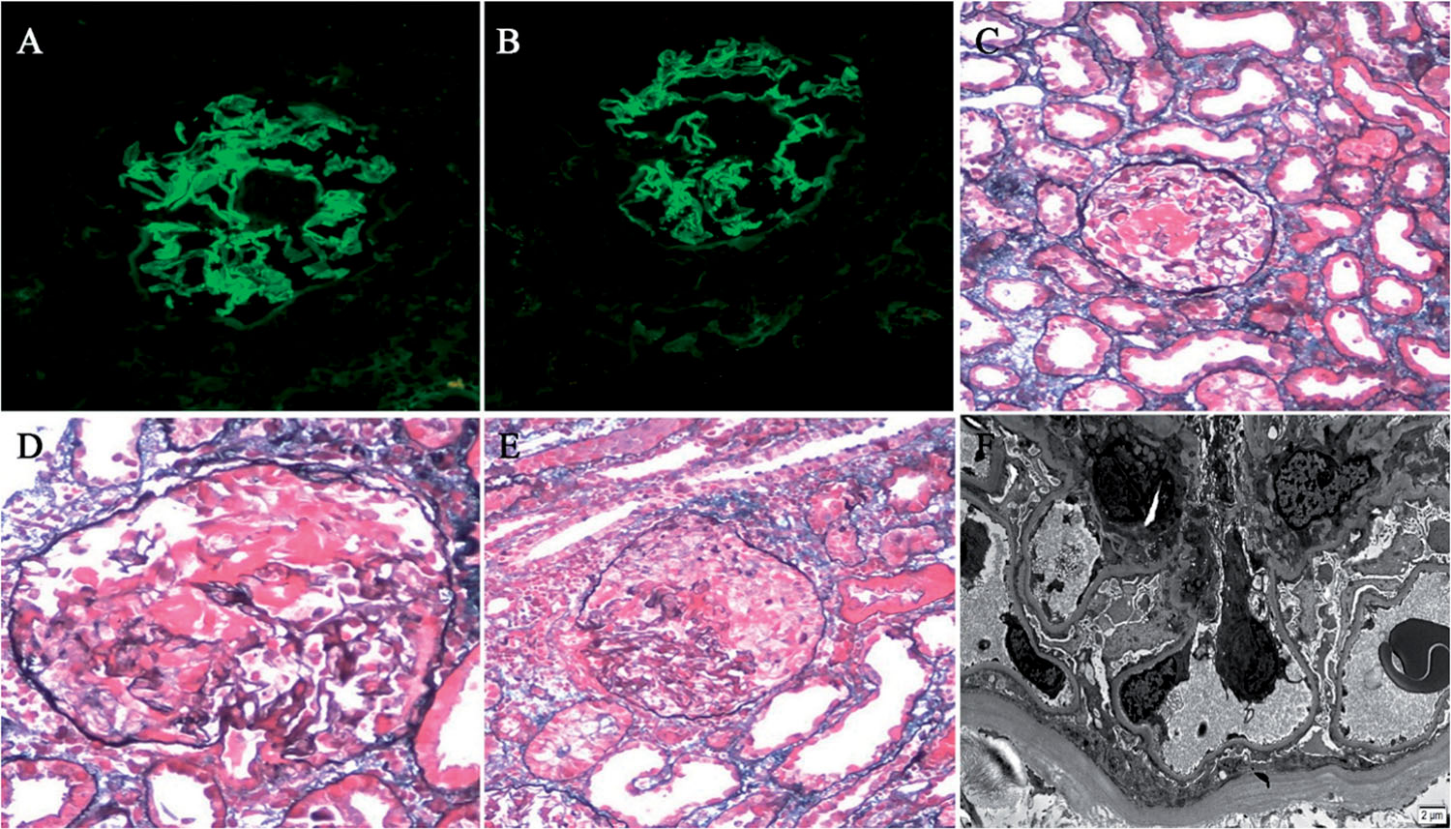
Diagnosis of anti-GBM disease mediated by IgG and IgA in the renal biopsy specimen. (A) Direct immunofluorescence analysis showed strong (2+ to 3+) linear capillary loop IgA (original magnification, ×200). (B) Direct immunofluorescence analysis showed strong (2+ to 3+) linear capillary loop IgG (original magnification, ×200). (C) Light microscopy showed segmental fibrinoid necrosis (PASM, ×200). (D) Light microscopy showed segmental fibrinoid necrosis (PASM, ×400). (E) Light microscopy showed fibrinoid necrosis with crescent formation (PASM, ×200). (F) Epithelial cell foot process fusion was detected, and no electron-dense deposits were found on electron microscopy (original magnification, ×5000).

Kidney biopsy was performed. Immunofluorescence analysis indicated bright capillary wall staining for IgA (2+ to 3+) (Figure 1(A)) and IgG (2+ to 3+) (Figure 1(B)). There was weakly capillary wall staining for κ, and λ (+), and segmental staining for IgM (1+) and FRA (2+). C3, C1q, HbsAg, HbcAg, and HCV-Ag were negative. All the four IgG subclassed deposition along GBM were detected. There was bright capillary wall staining for IgG1 (2+ to 3+) and IgG3 (2+), and weakly capillary wall staining for IgG2 (+) and IgG4 (+). Light microscopy showed segmental fibrinoid necrosis in five of 36 glomeruli (Figure 1(C,D)), and fibrinoid necrosis with crescent formation in seven of 36 glomeruli (Figure 1(E)). Epithelial cell foot process fusion was present, and no electron-dense deposits were found by electron microscopy (Figure 1(F)). The findings of renal biopsy suggested anti-GBM disease.

Informed consent was obtained for analysis of the patient’s serum. One human serum sample was used as a negative control, and another serum sample from an IgG anti-GBM patient was used as a positive control. Human kidney cortex basement membranes and non-collagenous (NC1) hexamers of type IV collagen were prepared as described previously [8–11]. Another commercial ELISA kit (EA 1251-9601 G; Euroimmun Medizinische Labordiagnostika (China), Beijing, China) using bovine kidney α3 (IV) collagen NC1 domain as an antigen was used to detect the presence of IgG anti-GBM autoantibodies in the patient’s serum. The recombinant human α3 (IV)NC1 (2 μg/L) were coated into the 96-well plates at 4 °C overnight. After blocking and washing, diluted blood samples (1:100) were added at 37 °C for 60 min. After washing, horseradish peroxidase (HRP)-conjugated mouse anti-human IgA antibodies (Sigma-Aldrich, St. Louis, MO) diluted at 1:5000 were added at 37 °C for 60 min. 3,3′,5,5′-Tetramethylbenzidine (TMB) liquid was applied as substrate and the color development was terminated by 1 mM sulfuric acid after 20 min. The plates were read at 450 nm and absorbance value of each sample was calculated. The patient’s serum contained IgA (82 RU/mL, normal range <20 RU/mL) and IgG (78 RU/mL, normal range <20 RU/mL) anti-GBM autoantibodies.

**Figure 2.**
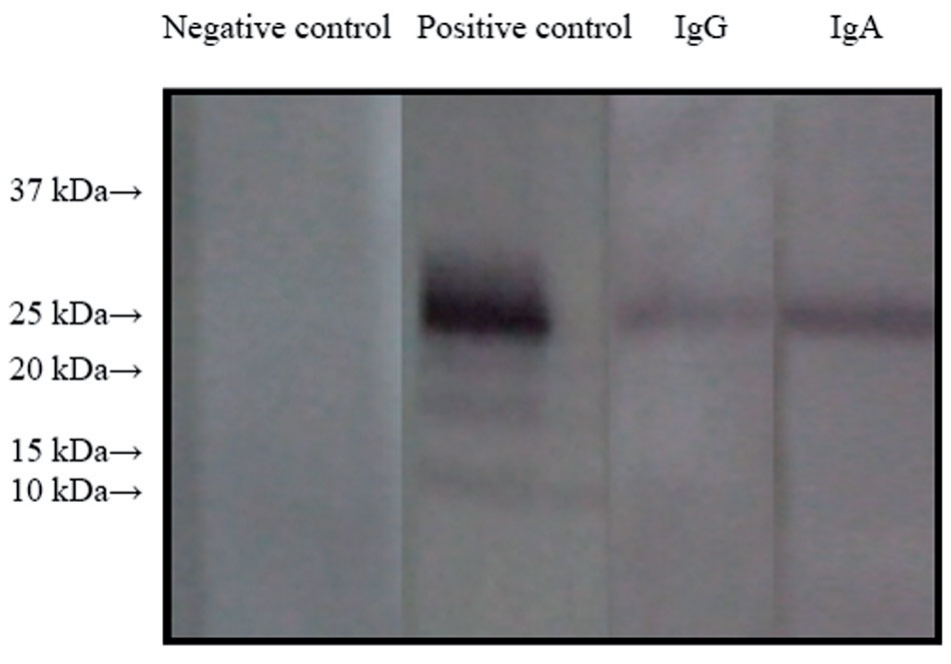
Western blotting analysis using purified human α(IV)NC1 as an antigen. Lane 1, negative control, serum from this patient + α5 (IV) collagen + rabbit anti-human IgG; lane 2, positive control, serum from an IgG anti-GBM GN patient + α3 (IV) collagen + rabbit anti-human IgG; lane 3, serum from this patient + α3 (IV) collagen + rabbit anti-human IgG; lane 4, serum from this patient + α3 (IV) collagen + rabbit anti-human IgA.

Further analysis was performed by Western blotting as described previously [12]. Briefly, recombinant human NC1 monomers were subjected to 12.5% sodium dodecyl sulfate (SDS)-polyacrylamide gel electrophoresis (SDS-PAGE) under non-reducing conditions and transferred onto a nitrocellulose membrane (Schleicher and Schuell, Kent, UK) using semi-dry blotting. The membrane was blocked in TBSTM buffer (0.01 mol/L Tris–HCl, pH 7.2, 0.15 mol/L NaCl, 0.1% Tween 20, 20 g/L skimmed milk) for 30 min at room temperature, incubated overnight with sera diluted 1:50 in TBSTM at 4 °C, washed, and incubated with alkaline phosphatase-conjugated secondary antibodies (mouse anti-human IgA antibodies and goat anti-human IgG; 1:6000; Sigma-Aldrich, St. Louis, MO) for 1 h at room temperature. Antibody binding was detected using nitro blue tetrazolium and 5-bromo-4-chloro-3-indolyl phosphate as a substrate (Sigma-Aldrich, St. Louis, MO). The patient’s IgA and IgG were shown to bind to the NC1 domain of GBM collagen IV. Both IgA and IgG mainly targeted a 25-kDa antigen (α3(IV)NC1) (Figure 2).

The patient was treated with three sessions of pulse methylprednisolone (500 mg/day × three days), followed by oral prednisone (1 mg/kg/day) and cyclophosphamide (1 mg/kg/day). Plasmapheresis was performed every other day for a total of six times. After 2 weeks of treatment, serum creatinine level had decreased from 310 μmol/L to 83.4 μmol/L. The urinary protein level decreased to 4.15 g/day, and albumin increased to 33 g/L. Two months later, renal function was stable (serum creatinine 88 μmol/L), urine protein had further decreased to 1.12 g/day, and albumin had increased to 43 g/L. The urine protein level decreased to 0.46 g/day with stable creatinine level after 4 months. Fortunately, she had normal renal function (serum creatinine 70 μmol/L) without proteinuria (0.07 g/day) at last follow-up (4 years later). The patient was satisfied with the therapeutic effect, and the written informed consent was obtained from her.

## Discussion

The diagnosis of anti-GBM disease requires demonstration of anti-GBM antibodies in either the serum or kidneys. A reliable, sensitive, and highly specific ELISA was detected, and high titers of antibodies are detected in almost all patients with anti-GBM disease at the time of active disease [13]. However, standard assays for circulating anti-GBM antibodies are designed to detect only IgG. Therefore, standard assays would yield a negative result for serum containing non-IgG anti-GBM antibodies. If other types of circulating antibodies, such as IgA or IgM are suspected, separate tests are required. As the accuracy of serological assays is variable, a kidney biopsy to confirm the diagnosis is recommended unless contraindicated [14]. The serum from this patient contained IgA and IgG anti-GBM autoantibodies at similar levels detectable by a commercial ELISA kit. In addition, the pathological findings also suggested anti-GBM disease.

Anti-GBM disease is a rare autoimmune disorder with an estimated incidence of <1 per million population [2]. The major class of anti-GBM autoantibody deposited along the GBM on kidney biopsies is IgG, with exceptionally rare reports of IgA or IgM classified as atypical anti-GBM disease [4]. Collagen IV is the main constituent of all basement membranes, a specialized form of extracellular matrix, which supports tissue integrity and plays roles in a number of key functions, including cell signaling, morphogenesis, and tissue regeneration [15]. Commonly, IgG anti-GBM antibodies bind the α3 subunit of the NC1 domain of type IV collagen (α3(IV)NC1). In addition to the ubiquitous autoantibodies against α3 NC1, distinct antibodies specific for the α5 NC1 domain have also been detected [16]. We performed a further analysis of the IgA and IgG antibodies capable of binding to α3(IV)NC1 in our patient.

However, the pathophysiology of anti-GBM disease mediated by IgG and IgA is not entirely understood. Among the known IgA or IgM mediated anti-GBM diseases, most of the patients had some immune diseases, including Henoch-Schonlein purpura, Crohn’s disease, and systemic lupus erythematosus; on the other hand, the autoantigen might prove more to be heterogeneous: it belonged to α5 and α6 chains of type IV collagen [13]. In our case, there was no associated immune disease and no exacerbating factors or triggers, such as smoking, hydrocarbon exposure, chemicals or heavy metal exposure, etc. Hence, this form of IgG and IgA-related condition should be considered a different disease.

At present, the standard therapy for anti-GBM disease is a combination of immunosuppression and plasma exchange. Poor renal outcome is commonly associated with high serum creatinine (>500 μmol/L), large numbers of glomerular crescents (50%) on renal biopsy, or a need for dialysis at presentation [17,18]. Fortunately, this patient did not have such poor prognostic factors, and achieved complete remission after intensive treatment. However, the patient presented RPGN and a nephrotic range of proteinuria, the electron microscopy showed a diffuse effacement of podocyte foot processes in preserved structured glomeruli. Is there any possibility of coexisted diseases as podocytopathy? Although there are relevant reports, minimal change disease superimposed on anti-GBM antibody positive glomerulonephritis [19,20]. It is only speculated by clinical and pathological characteristics. The mechanism is not clear, but the prognosis of these patients was better than that of typical anti-GBM disease. Therefore, from the available data, we cannot completely exclude the patient coexisted podocytopathy.

## Conclusions

To our knowledge, this is the first report of anti-GBM disease mediated by IgG and IgA. The patient’s IgG and IgA autoantibodies targeted α3(IV)NC1. Glomerular lesions were not significantly different from classical IgG anti-GBM disease or other atypical anti-GBM disease. The patient responded well to conventional therapy and plasma exchange. On the other hand, conventional anti-GBM assays used in clinical practice would detect only IgG antibodies, but it may cause some missed diagnosis. If the clinical presentation and histopathological findings are suggestive of atypical anti-GBM disease, alternative laboratory tests such as Western blotting analysis can be used to confirm the diagnosis.
